# The effect of COVID-19 during pregnancy on postpartum depression and mother-infant attachment

**DOI:** 10.1007/s11845-025-03928-1

**Published:** 2025-03-24

**Authors:** Canan Satır Özel, Reyhan Ayaz Bilir, Evrim Şenkal, Tahsin Gökhan Telatar, Abdulkadir Turgut

**Affiliations:** 1 Istanbul Medeniyet University, Faculty of Medicine, Goztepe Prof. Dr. Suleyman YALCIN City Hospital, Department of Obstetrics and Gynecology, Istanbul, Turkey; 2 Istanbul Medeniyet University, Department of Obstetrics and Gynecology, Department of Perinatology, Istanbul, Turkey; 3 Goztepe Prof. Dr. Suleyman Yalcin City Hospital, Department of Pediatrics, Istanbul, Turkey; 4 Recep Tayyip Erdogan University, Faculty of Medicine, Department of Public Health, Rize, Turkey; 5 Istanbul Medeniyet University, Faculty of Medicine, Department of Obstetrics and Gynecology, Istanbul, Turkey

**Keywords:** COVID-19, Mother-infant attachment, Postpartum depression, Pregnancy

## Abstract

**Background:**

The psychiatric impacts of the COVID-19 pandemic are well-documented; however, its effects during the postnatal period have been less explored.

**Aims:**

We aimed to investigate the effect of COVID-19 on mother-infant attachment and postnatal depression in pregnant women during the pandemic.

**Methods:**

The study group consisted of women with the diagnosis of ‘pregnancy and COVID-19’ after the 20th gestational week (*n* = 55). The control group included women who did not have a history of COVID-19 in their pregnancy or until the study date (*n* = 52). Edinburgh Postpartum Depression Scale (EPDS) and Postpartum Attachment Scale (PPAS) were administered to the participants. COVID-19 patients were grouped according to the WHO classification.

**Results:**

The mean EPDS score was higher in the study group than in the control group (9.55 (7.17) vs. 6.65 (6.72) (*P* = 0.006)). There was no difference between the groups in the number of individuals with depression (EPDS score > 13) and PPAS factors. The mean EPDS score was found to be higher in the hospitalized group (*P* = 0.025). The mean EPDS score in the group with moderate or severe disease was higher than mild disease group (16 (8.49) vs. 8.67 (6.57) (*P* = 0.039)), there was no difference in terms of PPAS.

**Conclusion:**

While the presence of COVID-19 during pregnancy has been associated with elevated postpartum depression scores in pandemic pregnant women, hospitalization of women who had COVID-19 during pregnancy and had at least one family member with moderate or more severe illness resulted in higher depression scores. The presence of COVID-19 during pregnancy does not affect mother-infant attachment. It is recommended that women with a history of COVID-19 during pregnancy be evaluated more carefully for postpartum depression.

## Introduction

Coronavirus Disease 2019 (COVID-19), which first caused an epidemic of pneumonia in China at the end of 2019, was declared as the “first pandemic caused by coronaviruses” by the WHO on March 11, 2020 [1]. On the day the pandemic was declared, the first case was detected in our country [2], marking the beginning of a new era. This era introduced new concerns, including transmission routes, protective measures, quarantine days, the number of cases, and death rates. In the medical literature, COVID-19 has become the most widely research topic. Pregnancy period, on the other hand, is distinctive for the course, follow-up and treatment of various diseases. For this reason, the effects of COVID-19 on physical and mental health of pregnant women, as well as on their postpartum period have been the subject of numerous studies.

Prenatal depression and anxiety rates increased significantly during the pandemic, with higher anxiety rates reported due to the uncertainty and chronic stress associated with the pandemic [3]. In a meta-analysis, depression was found in 15% of pregnant women in the pre-pandemic period, compared to 40.7% during the pandemic. Similarly, medium–high anxiety was present in 29% of women pre-pandemic, while it increased to 72% during the pandemic [4].

Untreated mental health disorders during pregnancy have been reported to adversely affect both pregnancy outcomes and postpartum mental health. Additionally, children born to these mothers may experience more cognitive and behavioral problems, and the risk of mental illness is higher for these children [5, 6]. This underscores the seriousness of maternal depression as a mental illness that affects not only the mother but also the fetus and child.

Despite extensive research described on anxiety and depression during pregnancy, studies on postpartum depression and mother-infant attachment remain relatively few. Additionally, the prevalence of postpartum depression was found to range from 13 to 43% in studies using a cut-off point of Edinburgh Postpartum Depression Scale (EPDS) > 13, with the overall prevelance calculated as 28% using different cut-off points [7]. In another meta-analysis, higher EPDS scores were statistically insignificant, while anxiety scores were significantly higher compared to the pre-pandemic period [8]. During the ongoing pandemic, there were active patients on the one hand, while the rehabilitation phase began on the other. This highlighted the need to evaluate and treat individuals for post-COVID complications. For optimal maternal and infant health, the well-being of the postnatal mother’s mental health is undoubtedly crucial.

This study aims to assess the impact of COVID-19 during pregnancy on postpartum depression and mother-infant attachment, considering disease severity, hospitalization status, and familial COVID-19 exposure as potential influencing factors.

## Methods

The study group included women with babies under the age of 1 who were followed up in our hospital after the 20th gestational week with the diagnosis of “pregnancy and COVID-19,” either outpatient or inpatient. The control group included women who were pregnant in a similar period, had babies under the age of 1, did not have COVID-19 during pregnancy or the postnatal period when the study was conducted, and had no family contact with a COVID-19 patient.

Exclusion criteria:

- High risk in aneuploidy screening
- Fetal anomaly
- Intrauterine or postnatal infant death before study
- Conditions requiring intensive care hospitalization in the neonatal period
- Mothers of babies diagnosed with any disease until the study period
- Maternal psychiatric disease
- Non-Turkish-speaking individuals
- Preeclampsia during pregnancy
- Women with conditions that may cause additional anxiety, such as diabetes, cholestasis, premature rupture of membranes, etc.
- Premature birth

Ethical approval was granted from the Ministry of Health’s COVID-19 Research and the hospital ethics committee (decision no: 2022/0274- date: 27.04.2022). The rules of the Declaration of Helsinki were followed. Patient records were accessed through the hospital automation system. Patient forms and questionnaires were completed via teleconference after the verbal consent was obtained from the participation. All participants were contacted between 01.05.2022 and 30.06.2022. Participants gave birth between 04.02.2021 and 17.04.2022. The study group had COVID-19 between 13.10.2020 and 29.12.2021.

The study group was classified as mild, moderate, severe, and critical COVID-19 categories according to the World Health Ogranisation (WHO) [9]. According to the WHO, mild disease refers to individuals with COVID-19 who do not have hypoxia or viral pneumonia. Moderate disease involves pneumonia patients with SpO_2_ > 90%, while severe disease includes pneumonia patients with SpO_2_ > 90% or a respiratory rate above 30 breaths per minute. Critical disease is defined as patients experiencing ARDS, sepsis, septic shock, and acute thrombosis. Patients who had COVID-19 (study group) and those who did not (control group) were compared in terms of postpartum infant attachment and postpartum depression. To this end, the ‘Edinburgh Postpartum Depression Scale’ and the ‘Postpartum Attachment Scale’ were administered to the participants. Afterwards, the study group was divided into subgroups based on disease stage, hospitalization, the presence of a patient in the family, and the disease stage of the family members, and comparisons were made.

- Edinburgh Postpartum Depression Scale (EPDS)

This scale was developed for screening purposes to asses the risk of depression in women during the postpartum period. It is a self-assessment scale consisting of 10 items that assess the individual’s psychological state over the past seven days [10]. Each item is rated a four-point Likert scale, with responses ranging from 0 to 3 (“Yes, always,” “Yes, often,” “No, not very often,” and “No, never”). The total score can range from 0 to 30, with higher scores indicating greater severity of depression. The Turkish version of the EPDS was used in the study [11], with a cut-off point of 13 [12].

- Postpartum Attachment Scale (PPAS)

This scale was developed to facilitate the early diagnosis of problems in the mother-infant relationship and is completed by the mother [13]. The scale utilizes a six-point Likert scale, with response options defined as “always,” “very often,” “often,” “sometimes,” “rarely,” and “never.” Items are rated on a scale from 0 to 5. The validated Turkish version of the scale was used in our study [14]. The scale consists of 25 items, 17 of which are reverse scored. The scale includes the following factors: “factor 1: attachment disorder” (12 items), “factor 2: rejection and ırritability” (7 items), “factor 3: tension about care” (4 items), and “factor 4: “risk of abuse” (2 items). Pathology in the mother-infant relationship is diagnosed based on to the cut-off points determined for four subscales. The cut-off points for the subscales are as follows: attachment disorder (factor 1) ≥ 12, rejection and ırritability (factor 2) ≥ 17, infant care anxiety (factor 3) ≥ 10, risk of abuse (factor 4) ≥ 3, and for the entire scale ≥ 26.

The minimum number of participants required for the study was calculated using G*Power 3.1.9.7 software. To determine the relationships between various variables in the case and control groups, the minimum sample size was calculated to be 82, with a 95% power level, an effect size of 0.4, an α error level of 0.05, and 1 degree of freedom.

### Statistical analyses

Data were analyzed using IBM SPSS Statistics for Windows (Armonk, NY, USA, IBM Corp.). Numerical data are presented as means and standard deviation, while categorical data are presented as frequencies and percentages. The relationships between categorical variables were assessed using the Chi-square test and Fisher’s exact test. The distribution characteristics of continuous data were evaluated using the Kolmogorov–Smirnov test and the Mann–Whitney *U* test was appiled to variables that did not meet normal distribution. In all statistical analyses, a significance level of *P* < 0.05 was considered statistically significant.

## Results

**Fig. 1:**
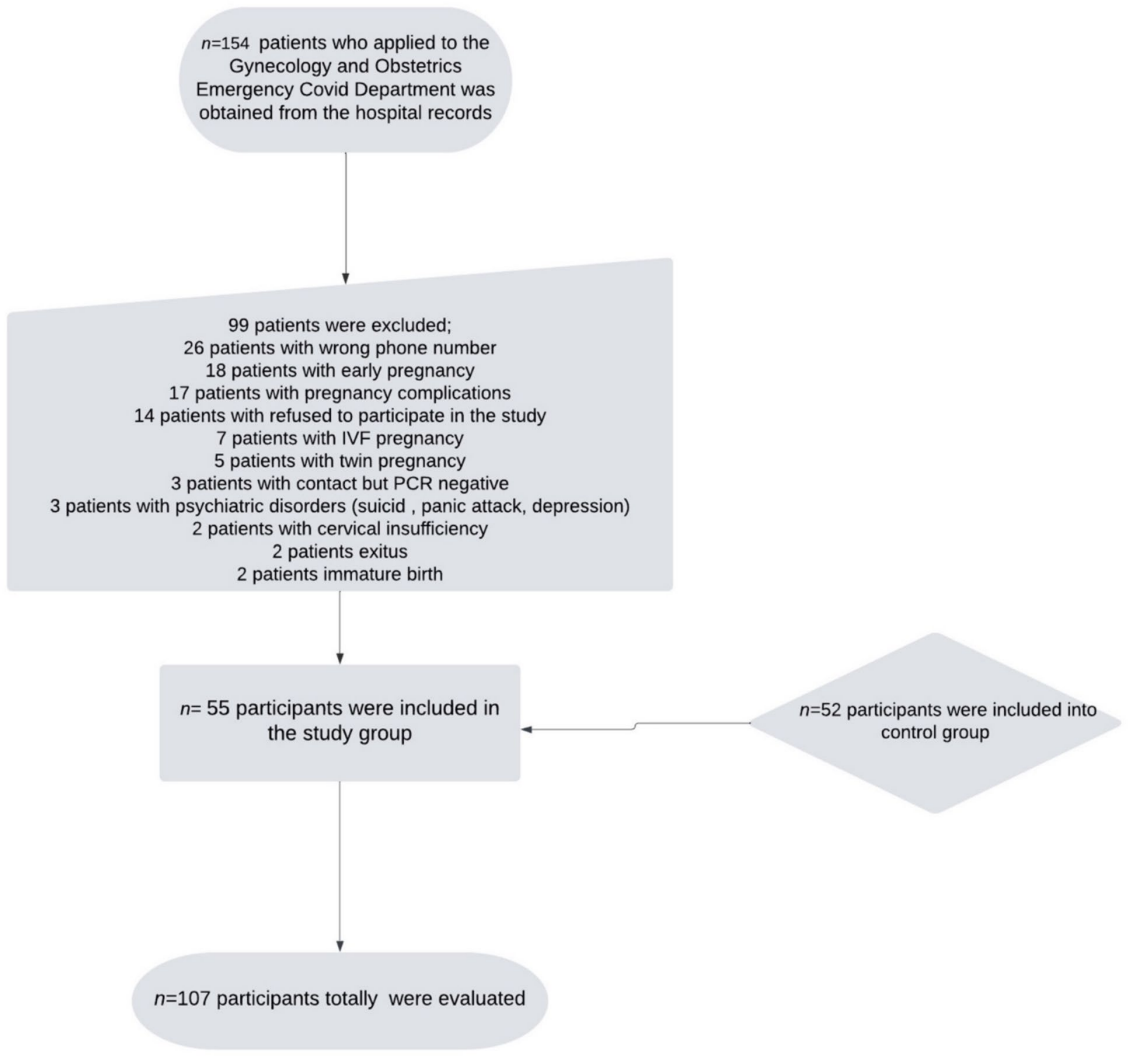
Study design

The admissions to the Obstetrics and Gynecology COVID-19 Emergency Department between October 1, 2020, and December 31, 2021, were reviewed using hospital records. A total of 154 pregnant women diagnosed with COVID-19 were admitted during this period. However, 99 patients were excluded for various reasons, and 55 patients were included in the study (Fig. 1). A control group consisting of 52 women who were pregnant and gave birth during the same period was formed. In total, 107 participants were included in the study.

The demographic data of participants are shown in Table 1. The groups were similar except for the mean gestational age at birth and the mean infant age. Additionally, in the COVID-19 group (study group), 24 patients were hospitalized, and 3 patients were followed up in the intensive care unit (ICU). Forty patients had Stage 1 COVID-19 disease. According to the EPDS, 21.5% of the participants were depressed. According to the PPAS, 7 participants had attachment disorder scores, 9 participants had anxiety about baby care, and 2 participants had abuse risk scores. In the study group, 39 patients had Stage 1, 11 patients had Stage 2, 2 patients had Stage 3, and 2 patients had Stage 4 disease.

**Table 1:** Demographic data of participants

	Control group (*n* = 52)	Study group (*n* = 55)	*P*
Mean mother age*	31.73 (5.74)	30.58 (5.18)	0.347
Mean gestational age of birth*	38.94 (1.2)	38.03 (2.15)	**0.034**
Mean infant age*	279.13 (95.21)	253.27 (93.26)	**0.049**
Pregnancy*	2.56 (1.35)	2.22 (1.17)	0.182
Parity*	2.06 (1.06)	1.93 (0.92)	0.472
Abortion*	0.5 (1)	0.24 (0.51)	0.234
Normal vaginal delivery*	0.73 (1.19)	0.87 (1.19)	0.482
Cesarian section*	1.37 (1.12)	1.07 (0.96)	0.191
Vaccine (least 1 dose)**			0.883
Yes	11 (21.15%)	11 (20.0%)	
No	41 (78.85%)	44 (80.0%)	
Delivery type**			0.816
Normal vaginal delivery	15 (28.85%)	17 (31.0%)	
Cesarian section	37 (71.15%)	38 (69.0%)	

^*^Mann–Whitney *U* test, mean (SD)

^**^Chi-square test, percentage

Table 2 shows the differences between the study and the control group. The mean birth week and infant age were statistically significantly lower in the study group, while the total EPDS score in the study group was 9.55 (7.17), it was 6.65 (6.72) in the control group, which was statistically significant (*P* = 0.006). In terms of the PPAS, no difference was observed between the groups.

**Table 2:** Differences between groups

	Control group 1 (*n* = 52)	Study group 2 (*n* = 55)	*P*
Mean gestational week, mean (SD)	38.94 (1.2)	38.03 (2.15)	0.034*
Mean postnatal infant age, mean (SD)	279.13 (95.21)	253.27 (93.26)	0.049*
Delivery type			
Normal vaginal delivery	15 (28.84%)	17 (31.0%)	0.816**
Cesarian section	37 (71.16%)	38 (61.0%)	
Mean EPDS score, mean (SD)	6.65 (6.72)	9.55 (7.17)	0.006*
Average number of depressed patients/number of patients according to Edinburgh			
Negative (< 13)	44 (84.62%)	40 (72.72%)	0.135**
Positive (≥ 13)	8 (15.38%)	15 (27.27%)	
Postpartum Attachment Scale, mean (SD)			
Mean of factor 1	4.46 (4.31)	3.31 (3.79)	0.153*
Mean of factor 2	1.25 (1.93)	0.84 (1.88)	0.098*
Mean of factor 3	1.87 (2.51)	1.53 (2.09)	0.676*
Mean of factor 4	0.13 (0.74)	0.16 (0.74)	0.462*
Number of patients factor 1 ≥ 12	4 (7.69%)	3 (5.45%)	0.711***
Number of patients factor 2 ≥ 17	-	-	-
Number of patients factor 3 ≥ 10	-	-	-
Number of patients factor 4 ≥ 3	1 (1.92%)	1 (1.81%)	0.999***

^*^Mann–Whitney *U* test

^**^Chi-square test

^***^Fisher’s exact test

The study group (*n* = 55) was compared according to the COVID-19 stage (‘Stage 1’ and ‘at least Stage 2’) and summarized in Table 3. The number of individuals who were not depressed (EPDS < 13) was 32 (80%) in the Stage 1 group (*n* = 40), and 8 (20%) in the least Stage 2 group (*n* = 15), and this was statistically significant (*P* = 0.048).

**Table 3:** Comparison of the Study Group in Subgroups According to COVID-19 Classification

	COVID-19 stage 1 (*n* = 40)	COVID**﻿-19** at least stage 2 (*n* = 15)	*P*
Mean age of infant age, mean (SD)	246.33 (95.64)	271.8 (86.97)	0.100*
Mean gestational week during COVID-19, mean (SD)	28.55 (8.10)	29.06 (6.48)	0.895*
Mean EPDS score, mean (SD)	8.28 (6.32)	12.93 (8.39)	0.062*
Average number of depressed patients/number of patients according to Edinburgh			
Negative (< 13)	32 (80.0%)	8 (53.34%)	0.048**
Positive (≥ 13)	8 (20.0%)	7 (46.66%)	
Postpartum Attachment Scale, mean (SD)			
Mean of factor 1	3.48 (4.24)	2.87 (2.23)	0.767*
Mean of factor 2	0.95 (1.99)	0.53 (1.6)	0.164*
Mean of factor 3	1.78 (2.25)	0.87 (1.46)	0.259*
Mean of factor 4	0.10 (0.38)	0.33 (1.29)	0.967*
Number of patients factor 1 ≥ 12	2 (5.0%)	1 (6.66%)	0.999***
Number of patients factor 2 ≥ 17	-	-	-
Number of patients factor 3 ≥ 10	-	-	-
Number of patients factor 4 ≥ 3	1 (2.5%)	0 (0.0%)	0.999***

^*^Mann–Whitney *U* test

^**^Chi-square test

^***^Fisher’s exact test

Individuals in the study group were evaluated in subgroups based on having at least one family member with COVID-19 (*n* = 49) and not having COVID-19 in the family (*n* = 6). No significant difference was observed in any of the parameters.

According to the COVID-19 stage of the group in which at least one of the family members is COVID-19; they were divided into subgroups: Stage 1 (*n* = 42) and at least Stage 2 (*n* = 7). While the total score of EPDS score was 16 (8.49) in the group with the least Stage 2 COVID-19 family members, the score in the Stage 1 group was 8.67 (6.57), and a statistically significant difference was found (*P* = 0.039). No significant difference was observed in the other parameters (Table 4).

**Table 4:** According to the COVID-19 stage of the family member in those who have at least one of the family members in the study group

	Group with all family members having COVID-19 disease stage 1 (*n* = 42)	Group with at least one family member of COVID-19 disease stage 2 and above (*n* = 7)	*P*
Mean age of infant age, mean (SD)	253.9 (101.25)	277.29 (44.87)	0.864*
Mean gestational week during COVID-19, mean (SD)	28.19 (7.92)	32.63 (3.2)	0.214*
Mean EPDS score, mean (SD)	8.67 (6.57)	16 (8.49)	0.039*
Average number of depressed patients/number of patients according to Edinburgh			
Negative (< 13)	32 (76.19%)	3 (42.85%)	0.071**
Positive (≥ 13)	10 (23.81%)	4 (57.14%)	
Postpartum Attachment Scale, mean (SD)			
Mean of factor 1	3.33 (4.2)	2.86 (0.9)	0.377*
Mean of factor 2	1 (2.08)	0.14 (0.38)	0.262*
Mean of factor 3	1.79 (2.2)	1.00 (1.83)	0.445*
Mean of factor 4	0.21 (0.84)	0.00 (0.00)	0.400*
Number of patients factor 1 ≥ 12	2 (4.76%)	0 (0%)	0.999***
Number of patients factor 2 ≥ 17	-	-	-
Number of patients factor 3 ≥ 10	-	-	-
Number of patients factor 4 ≥ 3	1 (2.38%)	0 (0%)	0.999***

^*^Mann–Whitney *U* test

^**^Chi-square test

^***^Fisher’s exact test

The individuals in the study group were divided into subgroups based on hospitalization due to COVID-19 and compared in Table 5. While 31 individuals were not hospitalized, 24 individuals were not hospitalized. The total EPDS score in hospitalized patients was found to be statistically significantly higher than in non-hospitalized patients (12.46 (8.33) vs. 7.29 (5.24), (*P* = 0.024)).

**Table 5:** Comparison of hospitalization among study groups

	Outpatient (*n* = 31)	Inpatient (*n* = 24)	*P*
Mean age of infant age, mean (SD)	240.55 (104.68)	269.71 (75.02)	0.396*
Mean gestational week during COVID-19, mean (SD)	27.57 (8.68)	30.14 (5.9)	0.406*
Mean EPDS score, mean (SD)	7.29 (5.24)	12.46 (8.33)	0.024*
Average number of depressed patients/number of patients according to Edinburgh			
Negative (< 13)	25 (80.64%)	15 (62.5%)	0.034**
Positive (≥ 13)	6 (19.36%)	9 (37.5%)	
Postpartum Attachment Scale, mean (SD)			
Mean of factor 1	3.42 (4.13)	3.17 (3.37)	0.932*
Mean of factor 2	0.77 (1.91)	0.92 (1.89)	0.791*
Mean of factor 3	2.13 (2.38)	0.75 (1.33)	0.026*
Mean of factor 4	0.13 (0.43)	0.21 (1.02)	0.474*
Number of patients factor 1 ≥ 12	2 (6.45%)	1 (4.16%)	0.999***
Number of patients factor 2 ≥ 17	-	-	-
Number of patients factor 3 ≥ 10	-	-	-
Number of patients factor 4 ≥ 3	0 (0%)	1 (4.16%)	0.436***

^*^Mann–Whitney *U* test

^**^Chi-square test

^***^Fisher’s exact test

## Discussion

Pregnant woman during the pandemic period who had COVID-19 during pregnancy were observed to have a higher average depression score according to the EPDS. Having a Stage 1 COVID-19 was associated with a higher number of participants who were not depressed according to EPDS, compared to those who had at least Stage 2 COVID-19. We observed that a history of hospitalization increased the mean EPDS score and the number of participants with depression. A history of at least one family member with COVID-19 family member had no effect on EPDS and PPAS, but having a family member with at least Stage 2 COVID-19 increased the total scores EPDS scores. Having COVID-19 during pregnancy, COVID-19 stage, history of hospitalization, and COVID-19 history and stage in family members had no effect on PPAS.

Postpartum depression (PPD) is a condition that develops during pregnancy or in the first year after delivery, lasts longer than 14 days, and significantly affects the woman’s life. In other words, PPD is defined as a depressive disorder that begins within the first 4 weeks after pregnancy or birth and lasts throughout the first year of life [12, 15–19]. Postpartum depression is redefined as “peripartum-onset depressive disorder” in the Diagnostic and Statistical Manual of Mental Disorders-5 (DSM-V) [19].

Signs and symptoms include mood changes, loss of appetite or overeating, fatigue, feelings of worthlessness, decreased interest in previously enjoyed activities, distancing from loved ones, irritability, fatigue, sleep problems, difficulty bonding with the baby, and concerns about harming oneself or the baby [15, 17]. In summary, both psychological and somatic symptoms are observed. However, the most important of these symptoms is the mother’s thoughts of harming herself or her baby [20]. The prevalence of postpartum depression in the perinatal period is 11.5% [21]. During the COVID-19 pandemic, the prevalence of PPD was calculated to be 34%. It is thought that perinatal depression develops through the interaction of genetic, epigenetic, neuroendocrine, environmental, and social factors [20].

Postpartum psychosis is an extremely rare emergency. Postpartum psychosis is classified as “Brief Psychotic Disorder” in DSM-V [21]. Disorganized behavior and psychotic symptoms are observed in these patients. Untreated depression during the postpartum period can cause intense sadness and anxiety in the mother, indifference towards the child, and, as a result, inadequate mother-infant attachment. The lactation period may also be shorter in these women, or they may refuse to breastfeed. If the depression is severe, the mother may harm herself or the baby, and suicide and infanticide may occur. Suicide is a significant factor responsible for 20% of all maternal deaths in the first year after birth [22]. While harming the baby occurs in 7% in non-depressed mothers, this rate may increase to 41% in depressed women [23]. Infants of mothers whose postpartum mood disorders were not treated may experience more impairments in cognitive, behavioral, and emotional development, as well as delays in social and communication skills [20].

In summary, perinatal depression is a significant condition that affects the health of the mother, the health of the baby, and their physical, psychosocial, and neurocognitive development. Obstetricians and gynecologists should be able to identify these patients during the perinatal period and refer them to psychiatrists when necessary. In their practice, it would be appropriate for them to at least evaluate the vulnerable population more carefully.

Caring for a baby is perhaps the hardest and most long-term care that people provide. It is a fact that people can show their babies more patience, effort and dedication than they can show even in the care of their parents. The greatest workload in this regard is primarily on the mother. For this to occur, mother-infant bonding is likely one of the most important protective factors for the continuation of the generation. Studies investigating mother-infant attachment have shown decreased gray matter volume in the anterior, posterior, cortical midline, bilateral prefrontal and temporal cortex, as well as an increase in parietal lobe and midbrain gray matter volume on magnetic resonance (MR) images [24]. Becoming a mother even changes a woman’s brain. Additionally, mother-infant attachment has positive effects on child development. Orphaned and vulnerable children experience 14.7% emotional problems, 34.9% behavioral problems, 8.6% hyperactivity, 15.8% peer problems, and 3.4% prosocial problems [25].

In many societies, family and relatives support during the perinatal period—spending time together, preparing for the new family member, and supporting the puerperal and the new father—is a good example of solidarity. However, this support decreased due to lockdowns in some countries. It is well known that the maternal bond can be strained or disrupted by prenatal stress, health risks, and postnatal anxiety [26].

In line with previous studies, The Center for Epidemiologic Studies’ Depression Scale (CES-D) and The Mother-to-Infant Bonding Scale (MIBS) were applied to 227 mothers after 35th week of pregnancy and on home visits during the 7–8th days postpartum. A negative relationship was found between the level of depression during pregnancy and postpartum and the mother-infant bond after delivery [27]. In a recent study, one hundred and forty mother-infant pairs were evaluated using the Edinburgh Postpartum Depression Scale (EPDS) and the Postpartum Bonding Questionnaire (PBQ). The psychiatric diagnosis of the parents also affected the PBQ, and successful treatment of depressive symptoms paralleled a significant reduction in attachment disorder [28]. In another study from our country, depression and mother-infant attachment scores were investigated among women who were not infected during the COVID-19 pandemic. Postpartum women were able to spend more time with their babies and their families due to visiting restrictions, and as a result, the rate of postpartum depression decreased [29].

While much of the available research explores the stress of an uncertain future during the pandemic, the social isolation specific to COVID-19, the distress under even ideal conditions, and the deterioration of maternal mental health and the mother-infant bond, our study focuses on the transmission and stage of COVID-19, hospitalization, and the impact of family members’ disease status on mothers, and consequently, on mother-infant attachment.

In another and recent study, 305 prepandemic and 298 pandemic participants were compared. There was no difference in maternal-infant attachment, but the risk of depression was 65% higher, and the risk of anxiety was 46% higher during the pandemic period [30]. Postpartum depression during the COVID-19 period was found to be 28% when EPDS > 13 was used [7]. In our study, when EPDS > 13 was used, it was observed at a rate of 15.38% in the group without a history of COVID-19 and 27.27% in those with a history of COVID-19. This was observed in 21.49% of the entire study population. Depression rates (as defined by EPDS > 13) were observed to be higher among individuals with COVID-19 positive relatives during the pandemic. Depression was observed in 80% of individuals with COVID-19 positive relatives, while it was observed in 31.7% of those with non-positive relatives [31].

A limitation of our study is that it was not conducted in the early stages of the pandemic. As individuals gained access to the vaccine and were vaccinated, as well as due to the increased knowledge about the disease, this may have contributed to lower rate of depression. Another limitation of the study is that the control group consisted of individuals and family members who were not infected with COVID-19 up until the time of the study. However, it is possible that some individuals within this group were infected but did not seek hospital care due to being asymptomatic. Futhermore, factors such as socioeconomic status, education level, and monthly income were not evaluated as potential confounding variables that could influence the psychological state of the patients.

The American College of Obstetricians and Gynecologists (ACOG) has reported that pregnant women should be evaluated for depression at least once during pregnancy and the postpartum period for [18]. The American Academy of Pediatrics (AAP) recommends screening mothers for postpartum depression (PPD) at 1, 2, 4, and 6 months postpartum [32].

Neuropsychiatric disorders are increasingly recognized as common consequences of viral infections, with their etiopathogenesis being a subject of ongoing investigation. In the case of COVID-19, SARS-CoV-2 infection triggers a cascade of molecular disturbances, including excessive cytokine release (IL-6, TNF-α, and IL-1β), microglial activation, and oxidative stress, leading to neuroinflammation and neuronal dysfunction. Dysregulation of serotonin and dopamine metabolism, mediated by disruptions in the tryptophan-kynurenine pathway and tyrosine hydroxylase (TH) activity, contributes to neurotransmitter imbalances associated with depression. Furthermore, N-methyl-D-aspartate (NMDA) receptor dysfunction, altered glutamate homeostasis, and hypothalamic–pituitary–adrenal (HPA) axis hyperactivity result in persistent neuropsychiatric impairments, underscoring the intricate relationship between viral pathophysiology and mental health disorders [33].

These findings underscore the importance of routine postpartum depression screening in pregnant women with a history of COVID-19 infection, particularly those with severe illness or prior hospitalization, while also highlighting the necessity of psychiatric evaluation in severe cases.

## Data Availability

The data that support the findings of this study are available from the corresponding author upon reasonable request.
